# Variability in Loss of Multiple Enzyme Activities Due to the Human Genetic Variation P284T Located in the Flexible Hinge Region of NADPH Cytochrome P450 Oxidoreductase

**DOI:** 10.3389/fphar.2019.01187

**Published:** 2019-10-15

**Authors:** Shaheena Parween, Maria Natalia Rojas Velazquez, Sameer S. Udhane, Norio Kagawa, Amit V. Pandey

**Affiliations:** ^1^Pediatric Endocrinology, Diabetology, and Metabolism, Department of Pediatrics, University Children’s Hospital Bern, Bern, Switzerland; ^2^Department of Biomedical Research, University of Bern, Bern, Switzerland; ^3^Instituto de Investigaciones en Ciencias de la Salud, Universidad Nacional de Asunción, San Lorenzo, Paraguay; ^4^School of Medicine, Nagoya University, Nagoya, Japan

**Keywords:** POR, P450 oxidoreductase, CYP2C9, CYP2C19, CYP3A4, CYP3A5, protein- protein interaction, cytochrome P450

## Abstract

Cytochromes P450 located in the endoplasmic reticulum require NADPH cytochrome P450 oxidoreductase (POR) for their catalytic activities. Mutations in POR cause multiple disorders in humans related to the biosynthesis of steroid hormones and also affect drug-metabolizing cytochrome P450 activities. Electron transfer in POR occurs from NADH to FAD to FMN, and the flexible hinge region in POR is essential for domain movements to bring the FAD and FMN close together for electron transfer. We tested the effect of variations in the hinge region of POR to check if the effects would be similar across all redox partners or there will be differences in activities. Here we are reporting the effects of a POR genetic variant P284T located in the hinge region of POR that is necessary for the domain movements and internal electron transfer between co-factors. Human wild-type and P284T mutant of POR and cytochrome P450 proteins were expressed in bacteria, purified, and reconstituted for enzyme assays. We found that for the P284T variant of POR, the cytochrome c reduction activity was reduced to 47% of the WT and MTT reduction was reduced to only 15% of the WT. No impact on ferricyanide reduction activity was observed, indicating intact direct electron transfer from FAD to ferricyanide, but a severe loss of CYP19A1 (aromatase) activity was observed (9% of WT). In the assays of drug-metabolizing cytochrome P450 enzymes, the P284T variant of POR showed 26% activity for CYP2C9, 44% activity for CYP2C19, 23% activity for CYP3A4, and 44% activity in CYP3A5 assays compared to the WT POR. These results indicate a severe effect on several cytochrome P450 activities due to the P284T variation in POR, which suggests a negative impact on both the steroid as well as drug metabolism in the individuals carrying this variation. The negative impact of P284T mutation in the hinge region of POR seems to be due to disruption of FAD to FMN electron transfer. These results further emphasize the importance of hinge region in POR for protein flexibility and electron transfer within POR as well as the interaction of POR with different redox partners.

## Introduction

**Figure 1:**
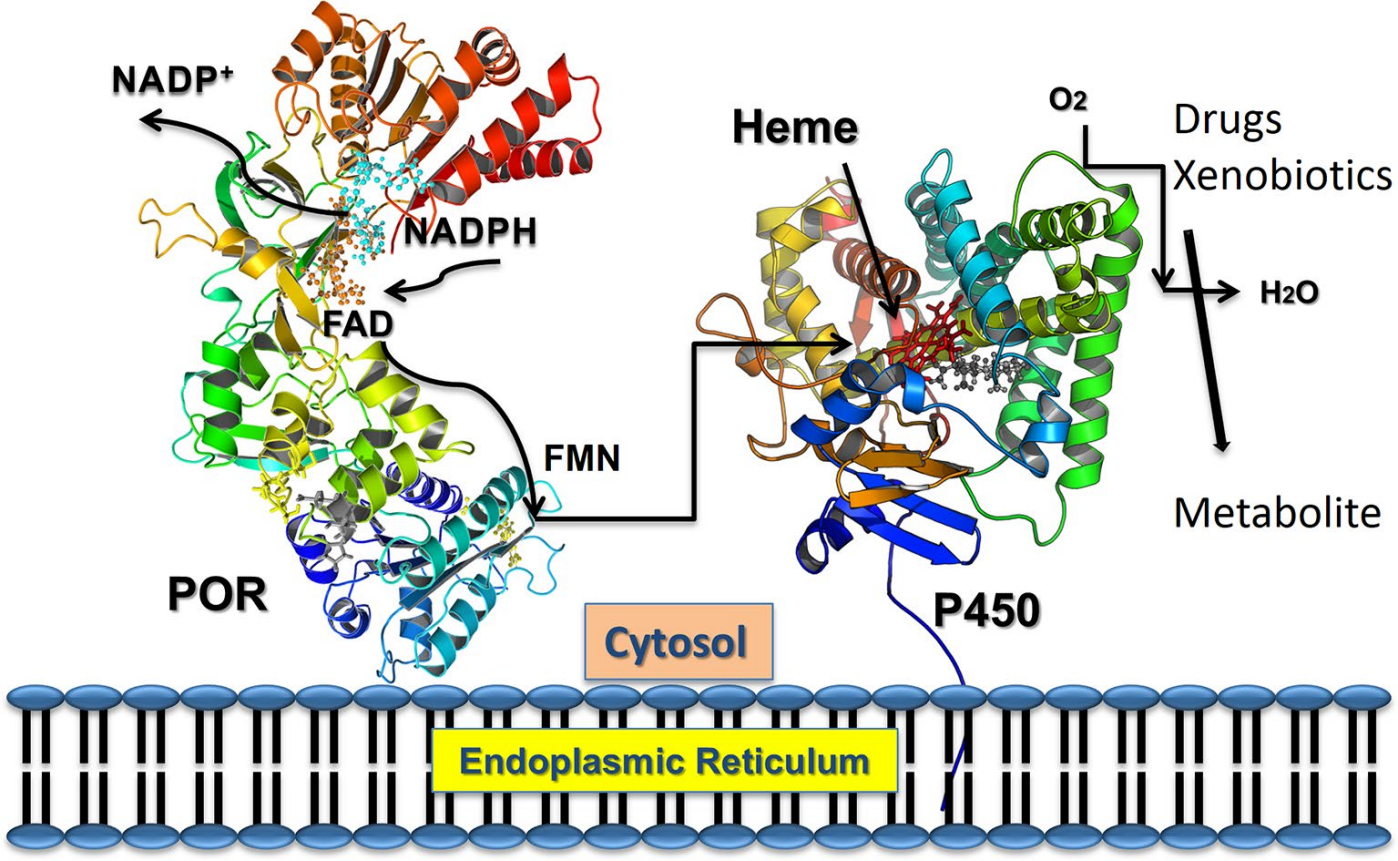
POR as electron transfer partner to cytochromes P450 proteins. Transfer of electrons from NADPH to redox partners of POR. Co-factor NADPH binds to the POR located into the endoplasmic reticulum and donates electrons which are received by FAD. Electron transfer to FAD causes a conformational change in POR that brings together the FAD, and FMN domains and electrons are then transferred from FAD to FMN. The FMN domain of POR interacts with the P450s and other redox partners and completes the final step of electron transfer. From Pandey, A. V. and Sproll, P. (2014). “Pharmacogenomics of human P450 oxidoreductase.” *Frontiers in Pharmacology* 5, 103.

The NADPH cytochrome P450 reductase (POR, NCBI# NP_000932, UniProt# P16435) is required for the enzymatic activities for all human cytochromes P450 proteins located in the endoplasmic reticulum (Pandey and Flück, 2013; Flück and Pandey, 2019). Cytochrome P450 proteins metabolize a broad range of endogenous and exogenous chemicals, including most of the drugs, steroids and other xenobiotics (Schuster and Bernhardt, 2007; Omura, 2010; Zanger and Schwab, 2013). There are two different types of cytochrome P450 proteins in mammals (Omura, 2010; Mclean et al., 2015). The type 1 P450s are located in the mitochondria and use the adrenodoxin/adrenodoxin reductase system as their redox partner (Omura, 2006; Zalewski et al., 2016). The type 2 cytochrome P450s are located in the endoplasmic reticulum and use a single diflavin reductase, POR, as their redox partner (Lu et al., 1969; Masters, 2005). POR contains both the flavin mononucleotide (FMN) and the flavin adenine dinucleotide (FAD), which are bound to distinct domains, and supplies electrons to cytochromes P450 and other proteins *via* protein-protein interactions (Iyanagi and Mason, 1973; Iyanagi et al., 1974; Matsubara et al., 1976; Vermilion and Coon, 1978; Oprian and Coon, 1980; Narayanasami et al., 1992) (**Figure 1**). The three-dimensional structure of the FMN binding domain of human POR has been solved by x-ray crystallography (Zhao et al., 1999) and more recently many structures of soluble POR proteins containing the NADPH, FAD, and FMN binding domains, but lacking the membrane-bound amino terminus sequence, have been determined by the groups of Kim and Masters (Wang et al., 1997; Xia et al., 2011a; Xia et al., 2011b). The FMN and FAD/NADPH binding domains of POR are connected by a flexible hinge region. The hinge region of POR is vital for domain movements within the POR protein and provides structural flexibility to bring the FAD and FMN domains together, in addition to influencing interactions with the redox partners of POR for electron transfer.

Human POR deficiency (PORD, OMIM: 613537 and 201750) is a form of congenital adrenal hyperplasia, initially described in patients with altered steroidogenesis reported by the laboratory of Walter Miller (Flück et al., 2004; Miller et al., 2004; Flück et al., 2007; Flück and Pandey, 2011; Flück and Pandey, 2013; Pandey and Flück, 2013; Fukami and Ogata, 2014; Pandey and Sproll, 2014; Flück and Pandey, 2019) followed by several reports with a broad spectrum of disorders (Adachi et al., 2004; Arlt et al., 2004; Pandey and Flück, 2013; Flück and Pandey, 2014; Pandey and Sproll, 2014; Oldani et al., 2015; Bonamichi et al., 2016; Nakanishi et al., 2016; Burkhard et al., 2017; Flück and Pandey, 2019). Congenital adrenal hyperplasia is an inborn error of adrenal steroid biosynthesis affecting the production of glucocorticoids (Miller and Fluck, 2014; Fluck, 2017). Defects in multiple steroid metabolizing cytochrome P450 proteins were suggested by Cedric Shackleton from the analysis of steroid hormones in the urine of patients with signs of disordered steroid metabolism, but no genetic mutations were identified (Peterson et al., 1985). Sequencing of the POR gene in patients with symptoms of mixed oxidase deficiency revealed mutations in POR linked to disorders of steroid biosynthesis (Flück et al., 2004; Miller et al., 2004; Pandey et al., 2004). Afterward, several different laboratories reported mutations in POR in patients with steroid biosynthesis disorders and bone malformation syndromes (Adachi et al., 2004; Arlt et al., 2004; Fukami et al., 2005; Pandey and Flück, 2013; Riddick et al., 2013).

In addition to steroid metabolizing cytochrome P450 enzymes, POR is also the redox partner of multiple drug metabolizing cytochrome P450 enzymes, and therefore, a negative impact of POR mutations is expected on activities of all partner proteins. While the initial studies on mutations in POR were aimed at deducing the impact on metabolism of steroid hormones, effects of POR mutations on other redox partners, including heme oxygenase and drug-metabolizing cytochromes P450, have been studied in later reports (Marohnic et al., 2006; Agrawal et al., 2008a; Agrawal et al., 2010; Flück et al., 2010; Marohnic et al., 2010; Nicolo et al., 2010; Pandey et al., 2010; Marohnic et al., 2011; Mccammon et al., 2016; Udhane et al., 2017; Velazquez et al., 2019). A mouse knockout (KO) model of *Por-/-* had been generated before the human mutations in POR were identified. The Por*-/-* was found to be embryonic lethal (Shen et al., 2002; Nicholas et al., 2017), and resembled the KO mouse model of retinoic acid-metabolizing *Cyp26a1-/-* (Gaston-Massuet et al., 2016). Liver-specific deletion of POR has been reported to cause lipidosis and increased liver weight (Gu et al., 2003; Wu et al., 2003). Deletion of *POR* gene in cardiomyocyte cells of the mouse leads to reduced P450 activity in the heart, but there was no indication of embryonic lethality or early morbidity and no difference in heart function was observed (Fang et al., 2008). A summary of the impact of *POR* variations and effects of *Por* knockout/knockdown had been provided by Riddick, Ding, Wolf Porter, and Pandey (Riddick et al., 2013).

Large-scale sequencing projects have now identified many variations in the *POR* gene in several different human subpopulations, and multiple POR variants have been linked to altered drug metabolism (Huang, 2008 #758; Gomes et al., 2009; Pandey, 2010 #579; Nicolo, 2010 #396; Agrawal, 2010 #720; Gomes et al., 2009; Velazquez, 2019 #2260). Several variants in the *POR* gene found in patients as well as non-clinical samples have been tested for enzymatic activities (Adachi et al., 2004; Arlt et al., 2004; Fukami et al., 2005; Fukami et al., 2006; Homma et al., 2006; Pandey, 2006; Huang et al., 2008a; Sim et al., 2009). While the mutations in POR could be located in all regions of the protein, based on analysis of previously identified mutations, some definite patterns have emerged. Mutations in POR that affect binding of co-factors (FMN/FAD/NADPH) cause a severe form of the disease with the mutations causing severe loss of FMN or FAD showing significant inhibitory effects on activities of all redox partners. We have previously shown that aromatase (CYP19A1) activity responsible for the metabolism of androgens is very susceptible to mutations in NADPH binding site of POR (Pandey et al., 2007; Flück et al., 2011; Flück and Pandey, 2017). On the other hand, mutations in POR affecting interaction with P450 and other redox partner are harder to predict since individual mutations may show different effects on individual partner enzymes.

While the mutations in the co-factor binding region of POR have clear mechanisms for loss of activities observed (explained by the reduction or loss of bound FAD or FMN), the mutations in the hinge region of POR require detailed characterization to understand the mechanisms of inactivation. While multiple mutations at or near the cofactor binding sites have been characterized in detail, mutations in the hinge region of POR have not received much attention. Considering the importance of the hinge region in the electron transfer process of the POR redox system, we looked at POR variants located in the flexible region of POR to understand the metabolic effects caused by mutations that may affect structural flexibility. In the current study, we have investigated the effects of POR variant P284T located near the hinge region of POR that is crucial for interactions with redox partner proteins, including P450s, to transfer electrons from NADPH. The POR variant P284T (rs72557937) was found in the Yoruba population through the sequencing of DNA obtained from a particular community in Ibadan, Nigeria (https://www.coriell.org/0/Sections/Collections/NHGRI/Yoruba.aspx?​PgId=128). Based on information available through the 1000 genome project, the samples were from parent-adult child trios, and all parents in the trios had identified themselves as having four Yoruba grandparents. The P284T variant of POR was also found in the African American population in the POR sequencing study carried out by Miller (Huang et al., 2008b). Previously, in a study based on computational prediction, we have identified structurally destabilizing population genetic variants in POR (Burkhard et al., 2017). In the analysis by Burkhardt et al. changes at the amino acid 284 of POR were predicted to cause protein malfunction (Burkhard et al., 2017). Here, we have tested the hypothesis that mutations in the hinge region of POR may cause loss of enzymatic activities that may depend on interactions with redox partners. In the current study, we aimed to characterize different activities of POR that are affected by the mutation P284T in the hinge region and assayed several drug-metabolizing cytochrome P450 enzymes, steroid metabolizing enzyme aromatase, and some direct reactions of POR like the reduction of small molecules. Our results confirmed that indeed, some reactions were unaffected by the P284T (Ferricyanide reduction activity), others had a range of effects from loss of 75–80% activity in case of CYP2C9 and CYP3A4, to CYP2C19, CYP3A5, and cytochrome c reduction that were only mildly affected. Aromatase, which requires several interactions with POR to complete one reaction cycle, was severely affected, losing more than 90% of the activity compared to reaction supported by the WT POR. These results indicate the importance of the hinge region in POR in the interaction with redox partners and confirm that changes in the hinge region may have variable effects on POR activity depending on individual redox partners.

## Materials and Methods

### Recombinant Expression of POR and Membrane Purification

The human POR WT and variant forms of proteins (NCBI# NP_000932, Uniprot# P16435) were recombinantly expressed in bacteria using the previously described methods and expression constructs (Huang et al., 2005; Pandey et al., 2007; Huang et al., 2008a; Nicolo et al., 2010). The protocol for expression of an N-27 form of POR variants and subsequent membrane purification have been adopted from our previous publications (Huang et al., 2005; Pandey et al., 2007; Pandey et al., 2010; Parween et al., 2016; Flück and Pandey, 2017). The cDNAs for WT or mutant POR in a pET22b vector were transformed into Escherichia coli BL21(DE3). Single colonies were selected for growth on ampicillin and grown in terrific broth pH 7.4 supplemented with 40 mM FeCl_3_, 4 mM ZnCl_2_, 2 mM CoCl_2_, 2 mM Na_2_MoO_4_, 2 mM CaCl_2_, 2 mM CuCl_2_, 2 mM H_3_BO_3_, 0.5 mg/ml riboflavin, 100 µg/ml carbenicillin at 37°C to an optical density (OD) 600 nm of 0.6 and temperature was reduced to 25°C for 16 h. The bacterial cells were collected by centrifugation, washed with PBS and suspended in 100 mM Tris-acetate (pH 7.6), 0.5 M sucrose, and 1 mM EDTA and treated with lysozyme (0.5 mg/ml) and EDTA [0.1 mM (pH 8.0)] at 4°C for 1 h with slow stirring to generate spheroplasts. The spheroplasts were pelleted by centrifugation at 5,000×g for 15 min; and suspended in 100 mM potassium phosphate (pH 7.6), 6 mM Magnesium acetate, 0.1 mM DTT, 20% (v/v) glycerol, 0.2 mM PMSF, and 0.1 mM DNase I; and disrupted by sonication. A clear lysate devoid of cellular debris was obtained by centrifugation at 12,000×g for 10 min, and then the membranes were collected by centrifugation at 100,000×g for 60 min at 4°C. Membranes containing POR were mixed in 50 mM Potassium phosphate buffer (pH 7.8) and 20% (v/v) glycerol and kept at −70ºC. The concentrations of proteins were measured by the RC-DC protein assay method (Protein Assay Dye Reagent, Bio-Rad, Hercules, CA) and POR content in membrane proteins was measured by western blot analysis.

### Western Blot Analysis of POR Content in the Bacterial Membranes

For Western blots, 1 μg of POR-WT and POR-P284T bacterial membrane proteins were separated on an SDS-PAGE gel and blotted on to polyvinyl difluoride (PVDF) membranes. Blots were first incubated with a rabbit polyclonal antibody against wild-type human POR from Genscript (Genscript, NJ, USA) at a dilution of 1:1,000. We then used a secondary goat anti-rabbit antibody labeled with a phthalocyanine infrared dye (IRDye 700DX, LI-COR Bioscience Inc., NE, USA) at a 1:10,000 dilution. Signals were analyzed with the green fluorescent channel (700 nm) on an Odyssey Infrared Imaging System (LI-COR Bioscience Inc., NE, USA), and protein bands were measured using the Odyssey software (LI-COR Bioscience Inc., NE, USA). POR content of each membrane preparation was measured, and all samples were normalized against purified wild-type POR used as a standard. In all experiments described in this report, the normalized amount of POR content was used for the mutants as well as WT POR protein.

### Small Molecule Reduction Assay by WT and POR-P284T

Assay of cytochrome c reduction by bacterially expressed WT or POR-284T was performed as described previously by measuring the change in absorbance at 550 nm (ε = 21.1 cm−1 mM−1) (Guengerich et al., 2009). In brief, the POR reduction reaction was performed in 96-well plates, in triplicate, with 5 µg membrane preparation containing POR in each well in 100 mM Tris-HCl (pH 7.5), using a microplate reader (Spectramax M2e, Molecular Devices, Sunnyvale, CA). The concentration of NADPH used was 100 µM and concentrations of cytochrome c (1.3–40 μM) were varied for kinetic analysis. The POR reduction reaction was initiated by the addition of NADPH, and the change in absorbance at 550 nm was monitored over 6 min. Data were fitted based on Michaelis-Menten kinetics (Michaelis and Menten, 1913) using GraphPad Prism (GraphPad Software, La Jolla, CA USA) to determine the Vmax and Km.

The NADPH-dependent MTT [3-(4,5-dimethylthiazol-2-yl)-2,5-diphenyltetrazolium] reduction rate was measured as the rate of increase in absorbance at 610 nm using an extinction coefficient of ε610 = 11 mM-1 cm-1 (Yim et al., 2005). The assay mixture contained 5 µg of bacterial membranes containing POR in 100 mM phosphate buffer (pH 7.6), 100µM NADPH and concentration of MTT varied from 3.9-500 µM. Similarly, the ferricyanide reduction rate was measured as the rate of decrease in absorbance at 420 nm (ε420 = 1.02 mM-1 cm-1). The concentration of ferricyanide varied over a range from 3.9 to 500 µM, and the reactions were started by adding 100µM NADPH (Marohnic et al., 2010). Activities represent the mean of at least triplicate determinations.

### Flavin Content Analysis of WT and Mutant POR

Protein-bound flavin molecules were released by thermal denaturation of POR proteins (Faeder and Siegel, 1973). Flavin content of WT and mutant POR proteins (100 µg/ml) was determined by boiling protein samples at 95°C for 10 min in the dark, followed by centrifugation at 14,000 ×g for 10 min to remove coagulated protein. The FMN and FAD ratio was determined by measurement of fluorescence of the supernatant at pH 7.7 and pH 2.6 (excitation at 450 nm, emission at 535 nm) (Faeder and Siegel, 1973).

### Expression and Purification of Human CYP19A1

The vector for bacterial expression of human CYP19A1was transformed in *E. coli* BL21(DE3) cells and the recombinant protein was expressed and purified following previously published protocols (Kagawa et al., 2003; Kagawa, 2011), with slight modifications. Briefly, a single transformed colony was selected for protein expression at 25°C. After 4 h of incubation, 1 mM δ-aminolevulinic acid (a heme precursor) and 4 mg/ml arabinose (for induction of molecular chaperones GroEL/GroES) were added to the culture and further incubated for 20 h. Cells were harvested, and spheroplasts were prepared with 0.2 mg/ml lysozyme in 50 mM Tris-Acetate (pH 7.6), 250 mM sucrose, and 0.5 mM EDTA at stored at -80°C. For protein purification, spheroplasts were lysed using 10XCellLytic B (Sigma-Aldrich) in buffer containing 100 mM potassium phosphate (pH 7.4), 500 mM sodium acetate, 0.1 mM EDTA, 0.1 mM DTT, 20% glycerol, and 1 mM PMSF. The cell lysate was centrifuged, and the supernatants were pooled for purification by Ni2+ affinity chromatography. Purification was performed at 4°C and protein concentration after the dialysis was determined by DC protein assay (Protein Assay Dye Reagent, Bio-Rad, Hercules, CA) using BSA as standard.

### Assay of Cytochrome P450 CYP19A1 in Reconstituted Liposome System

Purified recombinant CYP19A1 using the bacterial expression system was used to test the effect of POR-284T to support the aromatase activity of CYP19A1. Standard tritiated water release assay for the CYP19A1 activity was performed in a reconstituted liposome system using androstenedione as a substrate. Bacterial membranes containing POR and purified CYP19A1 were reconstituted into DLPC-DLPG liposomes. The liposomes were prepared previously (Udhane et al., 2017), and aromatase activity was measured by the tritiated water release assay originally described by Lephart and Simpson (Lephart and Simpson, 1991) using androstenedione as the substrate. Reaction mixture consisted of 100 pmol of CYP19A1, 400 pmol of POR, 100 mM NaCl and ^3^H labeled androstenedione ([1β-3H(N)]-andros-tene-3,17-dione; ∼20,000 cpm) in 100 mM potassium phosphate buffer (pH 7.4). Different concentrations (10–1000 nM) of androstenedione were used for kinetic analysis. The catalytic reaction was initiated by the addition of 1 mM NADPH, and the reaction tube was incubated for 1 h under shaking. Data were fitted based on Michaelis-Menten kinetics using GraphPad Prism (GraphPad Software, La Jolla, CA, USA).

### Assay of Cytochrome P450 CYP2C9 Activity in Reconstituted System

The activity of CYP2C9 promoted by WT or mutant POR was tested using the fluorogenic substrate BOMCC (Invitrogen Corp, Carlsbad, CA, USA). The purified CYP2C9 (CYPEX, Dundee, Scotland, United Kingdom) was used to test the activities of the POR variants using 20 µM BOMCC as substrate. *In vitro* CYP2C9 assays were performed using a reconstituted liposome system consisting of WT/mutant POR, CYP2C9, and cytochrome b_5_ at a ratio of 5:1:1. The final assay mixture consisted of 5 µg DLPC (1,2-Dilauroyl-sn-glycero-3-phosphocholine) and proteins (1 µM POR: 200 nM CYP2C9: 200 nM b_5_), 3 mM MgCl_2_, 20 µM BOMCC in 100 mM Tris-HCl buffer PH 7.4, and the reaction volume was 100 µL. The P450 reaction was started by addition of NADPH to a final concentration of 1 mM, and fluorescence was measured on a Spectramax M2e plate reader (Molecular Devices, Sunnyvale, CA, USA) at an excitation wavelength of 415 nm and an emission wavelength of 460 nm for BOMCC.

### Assay of Cytochrome P450 CYP2C19 Activity in Reconstituted System

The activity of CYP2C19 promoted by WT or mutant POR was tested using the fluorogenic substrate EOMCC (Invitrogen Corp, Carlsbad, CA, USA). The purified CYP2C19 (CYPEX, Dundee, Scotland, UK) was used to test the activities of the POR variants using 20 µM EOMCC as substrate. *In vitro* CYP2C19 assays were performed using a reconstituted liposome system consisting of WT/mutant POR, CYP2C9 and cytochrome b5 at a ratio of 5:1:1. The final assay mixture consisted of 2.5 µg DLPC (1,2-Dilauroyl-sn-glycero-3-phosphocholine) and proteins (0.5 µM POR: 100 nM CYP2C9: 100 nM b_5_), 3 mM MgCl_2_, 20 µM EOMCC in 100 mM Tris-HCl buffer PH 7.4 and the reaction volume was 100 µL. The P450 reaction was started by addition of NADPH to a final concentration of 0.5 mM, and fluorescence was measured on a Spectramax M2e plate reader (Molecular Devices, Sunnyvale, CA, USA) at an excitation wavelength of 415 nm and an emission wavelength of 460 nm for BOMCC.

### Assay of Cytochrome P450 CYP3A4 Activity in Reconstituted System

To compare the activities of CYP19A1 with another steroid-binding cytochrome P450, we tested the ability of POR mutations to support the enzyme activity of CYP3A4. The activity of the drug-metabolizing enzyme CYP3A4 supported by POR-WT or POR-284T was tested using the fluorogenic substrates BOMCC (7-Benzyloxy-4-trifluoromethylcoumarin) (Invitrogen Corp, Carlsbad, CA) as described earlier (Flück et al., 2010). The purified CYP3A4 (CYPEX, Dundee, Scotland, UK) was used to test the activities of the POR variants using 20 µM BOMCC. *In-vitro* CYP3A4 assays were performed using a reconstituted liposome system consisting of WT/mutant POR, CYP3A4, and cytochrome b_5_ at a ratio of 4:1:1 (POR : CYP3A4:b5). The final assay mixture consisted of liposomes and proteins (80 pmol POR: 20 pmol CYP3A4: 20 pmol b_5_), 2.5 mM MgCl_2_, 2.5 µM GSH and 20 µM BOMCC in 50 mM HEPES buffer and the reaction volume was 200 µl. The catalytic reaction was initiated by addition of NADPH to 1 mM final concentration, and fluorescence was monitored on a Spectramax M2e plate reader (Molecular Devices, Sunnyvale, CA) at an excitation wavelength of 415 nm and an emission wavelength of 460 nm.

### Assay of Cytochrome P450 CYP3A5 Activity in Reconstituted System

The activity of CYP3A5 promoted by WT or mutant POR was tested using the fluorogenic substrate BOMCC (Invitrogen Corp, Carlsbad, CA, USA). The purified CYP3A5 (CYPEX, Dundee, Scotland, UK) was used to test the activities of the POR variants using 20 µM BOMCC as substrate. *In vitro* CYP3A5 assays were performed using a reconstituted liposome system consisting of WT/mutant POR, CYP3A5 and cytochrome b_5_ at a ratio of 5:1:1. The final assay mixture consisted of 5 µg DLPC (1,2-Dilauroyl-sn-glycero-3-phosphocholine) and proteins (1 µM POR: 200 nM CYP2C9: 200 nM b5), 3 mM MgCl_2_, 20 µM BOMCC in 100 mM Tris-HCl buffer PH 7.4, and the reaction volume was 100 µL. The CYP3A5 reaction was started by addition of NADPH to 1 mM final concentration, and fluorescence was measured on a Spectramax M2e plate reader (Molecular Devices, Sunnyvale, CA, USA) at an excitation wavelength of 415 nm and an emission wavelength of 460 nm for BOMCC.

### Statistical Analysis of Results

Data are shown as mean, standard errors of the mean (SEM) in each group or replicates. Differences within the subsets of experiments were calculated using Student’s t-test. P values less than 0.05 were considered statistically significant.

## Results

### Effect of POR-P284T on Small Molecule Reduction Activity

**Figure 2:**
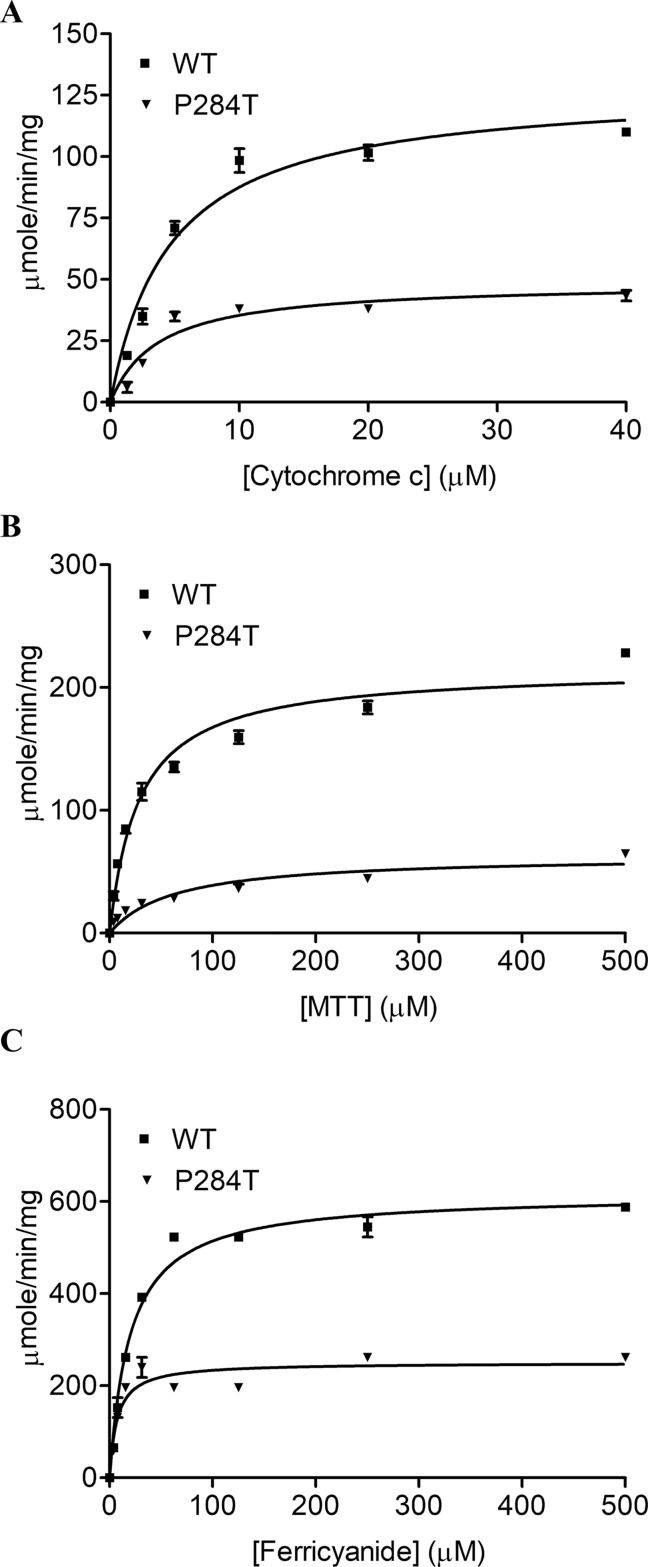
Cytochrome c, MTT, and Ferricyanide reduction assay with WT and POR-P284T. **(A)** Cytochrome c, **(B)** MTT and **(C)** ferricyanide reduction assays were performed with the WT and POR-P284T variant. Kinetic assays were performed by monitoring the changes in absorbance at 550 nm for cytochrome c, 610 nm for MTT, and 420 nm for ferricyanide reduction. Data were fitted to the Michaelis-Menten kinetics model and analyzed using GraphPad Prism. The calculated Km and Vmax values are presented in **Table 1**.

To study the effect of the P284T mutation on POR activity, we expressed WT and POR-P284T in *E. coli* and prepared the membranes. Using the membrane preparations containing the enriched POR, we measured their ability to transfer electrons from NADPH to cytochrome c, MTT, or ferricyanide. The POR-P284T mutation had a much lower capacity to reduce cytochrome c and MTT (**Table 1**, **Figures 2A, B**). Compared to WT POR, the P284T variant lost ∼50% of its activity to reduce cytochrome c and had only 15% of the WT POR activity to reduce MTT. However, the ferricyanide reduction activity (**Table 1**, **Figure 2C**) was not affected by the P284T mutation. Kinetic and statistical analysis is summarized in **Table 1**. The loss of activities with cytochrome c and MTT indicates disruption of electron transport from FAD to FMN, which could be due to conformational instability due to a P284T mutation affecting domain movements as well as a chain reaction starting from the disruption of electron transfer from NADPH to FAD. To differentiate between the two mechanisms, we tested the reduction of ferricyanide that can be reduced directly through FAD. Since the ferricyanide reduction activity of POR-P284T was comparable to WT, it can be deduced that electron transfer from NADPH to the FAD is not affected.

**Table 1:** Kinetic parameters for activities of cytochrome c, MTT, ferricyanide reduction, and CYP19A1 activity supported by POR-WT and POR-P284T variant.

	Km, (µM)	Vmax µmol/min/mg	Vmax/Km	% WT
***Cytochrome c reduction assay***				
WT	4.6 ± 0.7	128.2 ± 6.1	27.6	100
P284T	3.7 ± 0.8	48.7 ± 3	13	47
***MTT reduction assay***				
WT	19.9 ± 1.8	179.5 ± 4	9	100
P284T	48.3 ± 7.5	64.8 ± 3.1	1.3	15
***Ferricyanide reduction assay***				
WT	18.9 ± 2.4	600 ± 19	31.8	100
P284T	6.9 ± 1.6	248.1 ± 11	36	113
	Km, Androstenedione (nM)	Vmax pmol/min/nmol	Vmax/Km	% WT
***CYP19A1; aromatase (androstenedione to estrone)***				
WT	80 ± 14.5	0.72 ± 0.03	0.009	100
P284T	165.7 ± 147.9	0.12 ± 0.037	0.0008	9

For the cytochrome c, MTT and Ferricyanide reduction assay, the NADPH concentration was fixed at 100 µM and varying concentrations of the substrate were used for the analysis. For the conversion of androstenedione to estrone, NADPH was fixed at 1 mM, and variable concentrations (10–1000 nM) of androstenedione were used. Data are shown as mean ± SEM of independent replicates (n = 3 for cytochrome c, MTT and ferricyanide assay; n = 2 for CYP19A1 assay).

### The P284T Mutation in POR Causes a Reduction of Flavin Content

**Figure 3:**
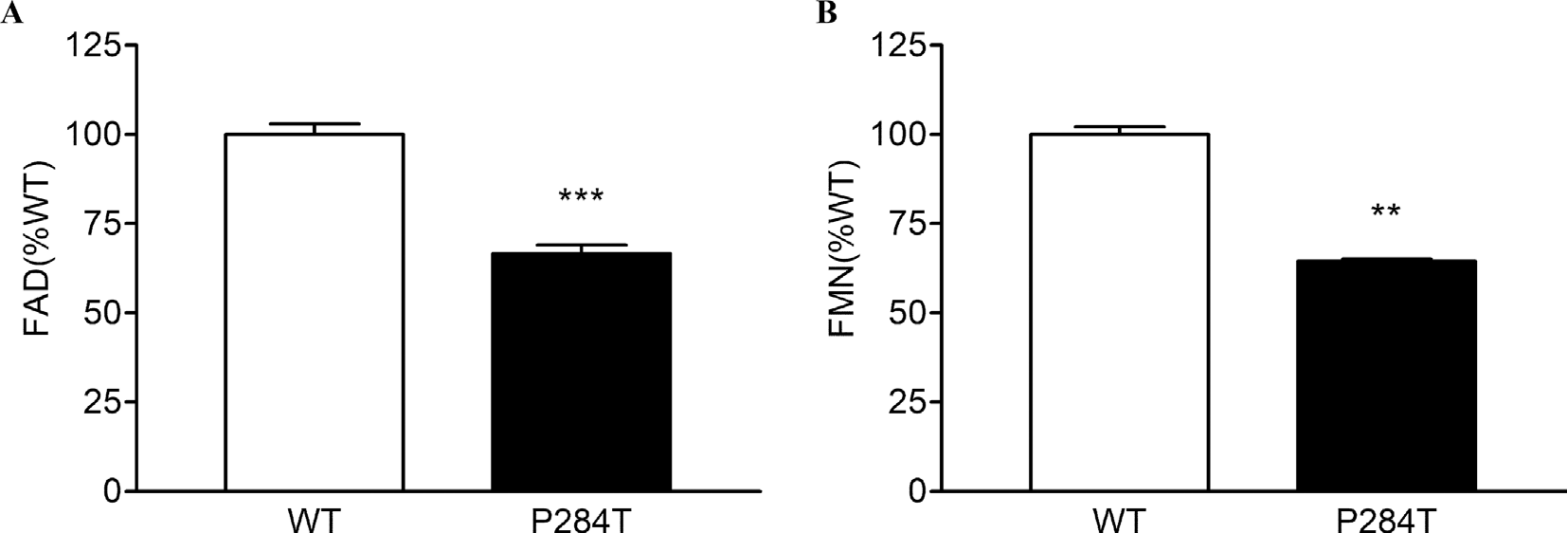
Flavin content of POR-WT and POR-P284T variant. Flavin content of the POR proteins was analyzed by boiling the proteins under selective pH conditions. Relative fluorescence unit (RFU) of the flavins released from the POR variants measured at **(A)** pH 2.6 (FAD or F_2.6_) and **(B)** pH 7.7 (FMN or F7.7) are shown. The RFU of POR-WT was fixed as a hundred percent. Data are shown as mean ± SEM of three independent replicates. Asterisks (**p < 0.01; ***p < 0.001) indicates significance based on students *t*-test.

To differentiate the conformational changes and effects of POR mutation P284T on flavin binding, we evaluated the relative flavin content since the activity of POR may be affected by the changes in the binding of cofactors FMN and FAD. As compared to WT POR, both the FMN and the FAD-binding was affected due to P284T mutation. The FMN content of POR-P284T was 64% as compared to WT, while the FAD content was reduced by 35%, suggesting that POR-P284T affects both FMN as well as FAD binding to POR (**Figure 3**). Differences compared to controls were calculated using Student’s t-test, and P values were below 0.01, indicating statistically significant differences. Since the residue P284 is not directly involved in either the FMN or the FAD-binding, a loss of flavins indicated a role of conformational changes caused by the P284T mutation to be responsible for the reduced flavin binding. The P284T mutation is, therefore, creating conformational changes that are less favorable for the binding of flavins in POR.

### CYP19A1-Aromatase Activity

**Figure 4:**
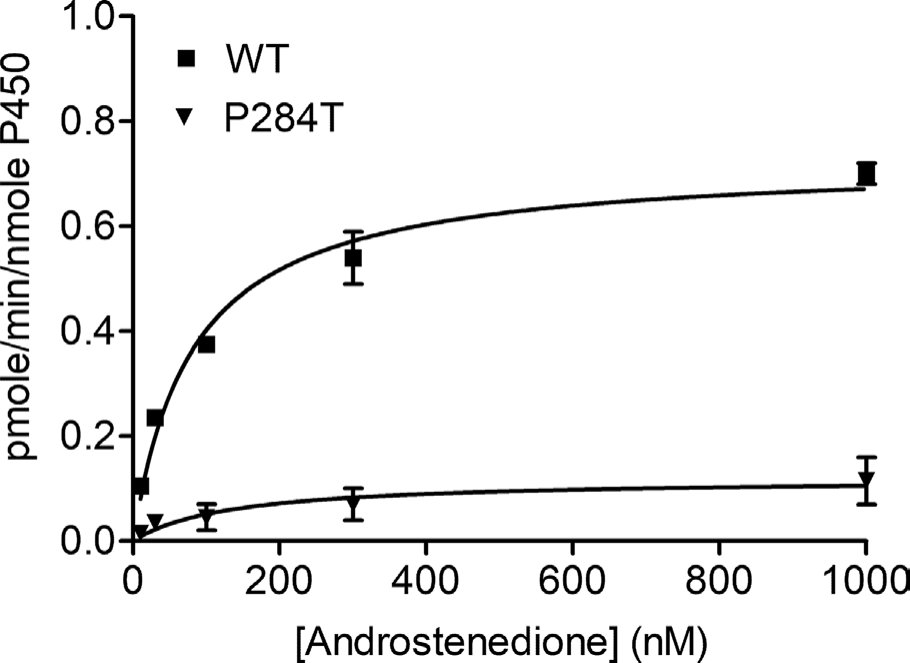
Enzymatic activity of CYP19A1 supported by POR-WT and POR-P284T variant. Bacterially expressed, purified, recombinant CYP19A1, and the enriched bacterial membranes containing POR proteins were mixed, and their activity to convert [3H] labeled androstenedione to estrone was tested by the tritiated water release assay. Data were analyzed using the Michaelis-Menten kinetics with GraphPad Prism. The calculated Km and Vmax values are shown in **Table 1**. Data are shown as mean ± SEM (n = 2).

The CYP19A1 requires six pairs of electrons for one reaction cycle, compared to two for most P450s, and therefore is more susceptible to changes in protein-protein interactions that dictate the electron transfer between POR and its redox partners. The POR-P284T showed almost complete loss of CYP19A1 activity (**Figure 4**, **Table 1**). For the POR-P284T variant, the apparent Km for androstenedione was increased by 2-fold as compared to WT POR suggesting that P284T mutation affects either substrate interaction with CYP19A1 or the CYP19A1-POR interaction. The apparent Vmax of POR-P284T was reduced by ∼85%. The POR variant P284T showed only 9% residual activity in supporting CYP19A1 as compared to WT POR. Kinetic and statistical analysis is summarized in **Table 1**.

### CYP2C9 Activity

**Figure 5:**
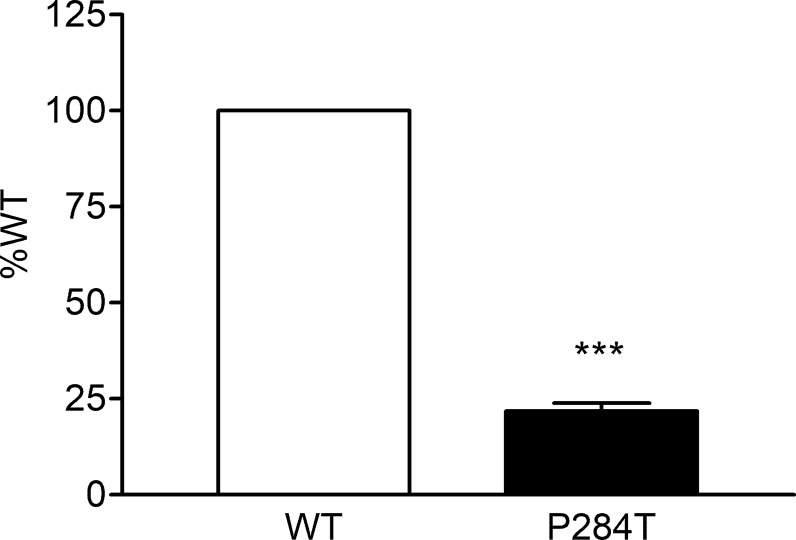
Activity of cytochrome P450 CYP2C9 supported by POR-WT and POR-P284T variant. Assay of CYP2C9 activity was performed to compare POR-WT and POR-P284T by using 20 µM BOMCC as a substrate. Activity with the WT POR was fixed as a hundred percent, and results are given as a percentage of WT activity. Data are shown as mean ± SEM of two experiments done in triplicates. ***p < 0.001 indicates significance based on students *t*-test.

We tested the activity of CYP2C9 supported by the WT and P284T variant of POR in reconstituted liposomes. Compared to WT POR activity of CYP2C19 supported by P284T variant of POR was reduced by 78% (**Figure 5**). This loss of more than three-quarters of activity compared to WT POR indicated a severe effect on drug metabolism supported by CYP2C9. Differences compared to controls were calculated using Student’s *t* **-**test, and P values were below 0.001, indicating statistically significant differences.

### CYP2C19 Activity

**Figure 6:**
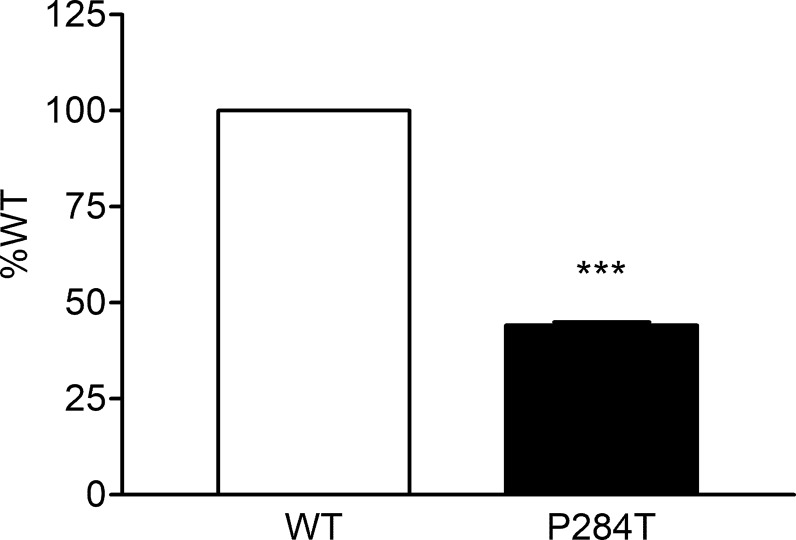
Activity of cytochrome P450 CYP2C19 supported by POR-WT and POR-P284T variant. Assay of CYP2C19 activity was performed to compare POR-WT and POR-P284T by using 20 µM EOMCC as a substrate. Activity with the WT POR was fixed as a hundred percent, and results are given as a percentage of WT activity. Data are shown as mean ± SEM of two experiments done in triplicates. ***p < 0.001 indicates significance based on students *t*-test.

The activity of CYP2C19 was tested with both the WT and P284T variant of POR. We found that in CYP2C19 assays, the P284T variant of POR showed only 44% of the WT POR activity with P values below 0.001 in Student’s t-test, indicating statistically significant differences (**Figure 6**). The effect of P284T variation in POR on CYP2C19 was not as strong as in case of CYP2C9 activity, but the loss of activity was still more than 50%, indicating a reduced capacity of drug metabolism reactions mediated by CYP2C19.

### CYP3A4 Activity

**Figure 7:**
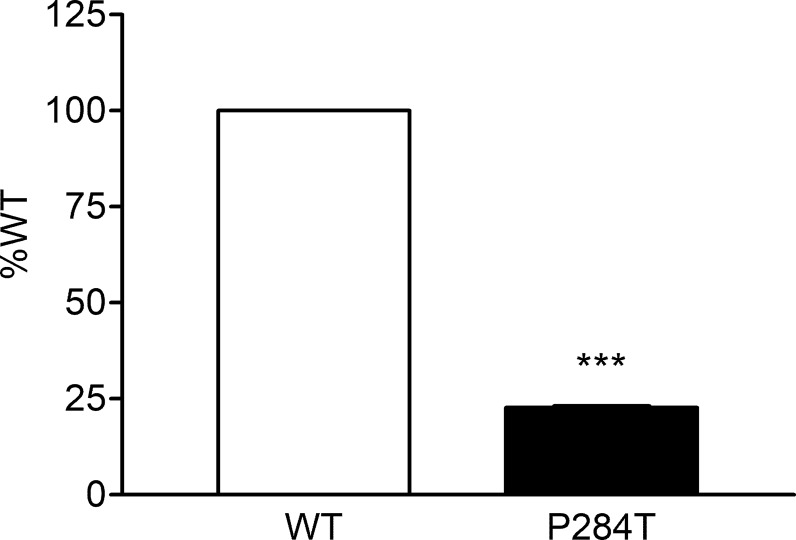
Activity of cytochrome P450 CYP3A4 supported by POR-WT and POR-P284T variant. Assay of CYP3A4 activity was performed to compare POR-WT and POR-P284T by using 20 µM BOMCC as a substrate. Activity with the WT POR was fixed as a hundred percent, and results are given as a percentage of WT activity. Data are shown as mean ± SEM of three independent replicates. ***p < 0.001 indicates significance based on students *t*-test.

The P284T variant of POR showed only 22.8% activity as compared to the WT enzyme with P values below 0.001 in Student’s t-test, indicating statistically significant differences (**Figure 7**). The loss of activities for drug-metabolizing cytochrome P450 enzyme-CYP3A4 by the POR variant P284T indicates problems with POR-P450 interactions which seem to be different for different cytochrome P450 partners.

### CYP3A5 Activity

**Figure 8:**
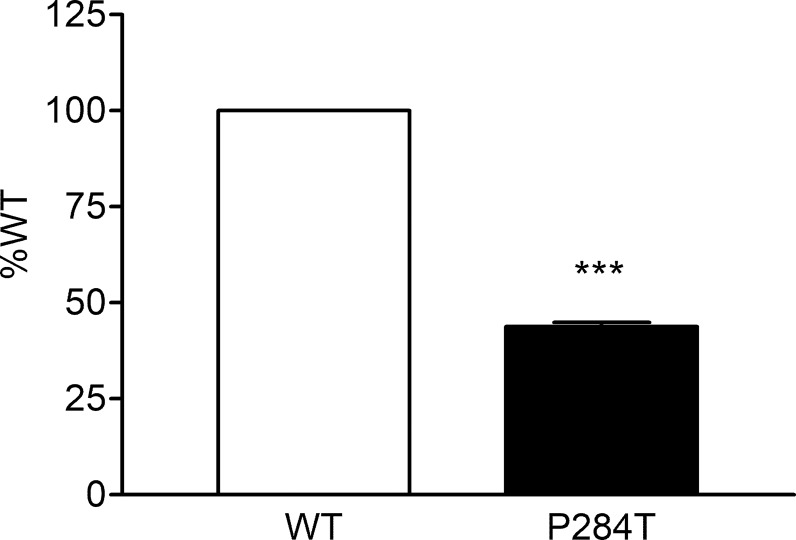
Activity of cytochrome P450 CYP3A5 supported by POR-WT and POR-P284T variant. Assay of CYP3A5 activity was performed to compare POR-WT and POR-P284T by using BOMCC as a substrate. Activity with the WT POR was fixed as a hundred percent, and results are given as a percentage of WT activity. Data are shown as mean ± SEM of two experiments done in triplicates. ***p < 0.001 indicates significance based on students *t*-test.

The 284T variant of POR had only 43.8% of the WT activity in CYP3A5 assay with P values below 0.001 in Student’s t-test, indicating statistically significant differences (**Figure 8**). This was different from the results obtained for the CYP3A4 activity assays, indicating there are differences in the interaction of POR with these two closely related cytochrome P450 proteins. The effect of R550W variation in POR on CYP3A5 was not as strong as in case of CYP3A4 activity, but the loss of activity was still more than 50%, indicating a reduced capacity of drug metabolism reactions mediated by CYP3A5. CYP3A5 is involved in the metabolism of tacrolimus, an immunosuppressant drug used during organ transplants which has a narrow therapeutic index.

## Discussion

In early reports, the effects of POR variants on drug-metabolizing cytochrome P450 enzymes has received less attention compared to effects on steroid metabolism (Scott et al., 2007; Agrawal et al., 2008b; Gomes et al., 2008; Huang et al., 2008b; Miller, 2012; Sahakitrungruang et al., 2009; Fukami, 2010). The cytochrome P450 subfamily 3A is the most versatile among all P450 enzymes and accounts for the metabolism of a large number of xenobiotics and pharmaceutical compounds. Cytochrome P450 3A4 (CYP3A4) is the principal hepatic enzyme that metabolizes a large percentage of drugs and endogenous substrates (Nicolo et al., 2010; Klein and Zanger, 2013; Meyer et al., 2013; Zanger and Schwab, 2013). CYP3A5 also has a significant role in xenobiotic metabolism in extrahepatic tissues like intestine and gut. The cytochrome P450 subfamily 2C is the second most relevant class of P450 proteins, and CYP2C9 and CYP2C19 are major drug-metabolizing enzymes.

Our result shows that mutations in POR can affect different activities of POR to varying extents, and some cytochrome P450 proteins are affected more than others. Also, there may be differences due to different allelic variations of cytochrome P450 proteins. The effects of different cytochrome P450 isoforms on the impact of POR activities has not been studied in detail. One study (Subramanian et al., 2012) tested different alleles of CYP2C9 (*CYP2C9.1*, *CYP2C9.2*, and *CYP2C9.3*) with a small number of POR variants but patterns were similar for all isoforms of CYP2C9 (Subramanian et al., 2012).

POR mutations leading to the significant loss of FAD/FMN co-factors hurt all redox partner activities (Marohnic et al., 2006; Marohnic et al., 2010; Nicolo et al., 2010). However, in most other cases, the effects of POR mutations on activities of the individual redox partner could be variable (Pandey and Sproll, 2014; Parween et al., 2016; Flück and Pandey, 2017; Udhane et al., 2017; Flück and Pandey, 2019). For the mutation P284T, we observed a small loss of cofactor binding, which could be due to overall protein stability changes due to the location of the P284 residue near the hinge region of POR that joins FMN binding domain with the FAD-binding domain. However, the activity of ferricyanide reduction, which represents direct electron transport through FAD without the involvement of FMN indicates that this loss of FAD did not severely reduce the overall quality of the protein. Cytochrome c reduction that involves protein-protein interaction and MTT reduction, which a dual electron transfer system through FMN showed loss of activities.

The location of the amino acid P284 in the hinge region indicated a potential impact on protein conformation and domain movements which may change protein-protein interaction, which may differ based on which redox partner is being tested. To study the role of amino acid P284 in POR on protein function and electron transfer in POR, we tested POR-P284T, a naturally identified mutation, with several different cytochrome P450 proteins. The CYP19A1 activity requires the transfer of three pairs of electrons, and therefore, is more susceptible to change in protein interaction with POR (Pandey et al., 2007; Flück and Pandey, 2017). A severe loss of CYP19A1 activity due to P284T mutation (9% of WT activity) indicated a significant effect on interaction with POR. In the case of drug-metabolizing cytochrome P450s, CYP2C9 and CYP3A4 lost more than 75% of activities while CYP2C19 and CYP3A5 lost more than 50% of the WT POR activity due to P284T mutation in POR.

On the other hand, there was no effect on the reduction of ferricyanide, a small molecule that can be reduced by direct electron transfer from FAD. This observation, together with loss of activity observed for reduction of MTT and P450 enzymes like CYP19A1, indicates that electron transfer from FAD to FMN is affected by changes in hinge region caused by P284T mutation. Taken together, these results indicate variability in interaction with different cytochrome P450s due to P284T mutation in POR. These results further underscore the fact that effects of POR mutations can be quite variable for different redox partners and mutations in POR require detailed characterization with different partner proteins and small molecule substrates to understand the biochemical consequences of individual POR mutations. These results also reinforce the idea that the hinge region of POR not only influences electron transfer within POR from FAD to FMN but also regulates the interaction of POR with different redox partners. In particular, we have shown here that a mutation in the hinge region of POR, P284T, changes the activity of a steroid metabolizing cytochrome P450 enzyme (aromatase) and several drug-metabolizing P450 enzymes.

Therefore, biochemical analysis using recombinant proteins is necessary to confirm the damaging effects of mutations with each redox partner separately (Parween et al., 2016; Udhane et al., 2017; Flück and Pandey, 2019). Several cytochrome P450 proteins for which POR is the redox partner, still have uncharacterized activities and their physiological roles are not defined. These other partners of POR could also influence the metabolic profiles of patients with PORD. Preliminary computational docking and functional analysis suggest that the loss of activity caused by some POR variants may be reversed by the introduction of external FMN (Nicolo et al., 2010) or FAD (Marohnic et al., 2006). However, the effect of flavin treatment in PORD needs to be tested in a clinical setting.

PORD changes both the steroid and the drug metabolism in the affected individual with varying effects. Interactions with redox partners are crucial for the functional electron transfer from POR. Different POR variations can cause highly variable effects on different metabolic reactions depending on the unique nature of the interaction of POR with different redox partners which depends on electrostatic interactions (Bernhardt et al., 1987; Bernhardt et al., 1988). A careful examination of affected target proteins by use of urine/serum steroid analysis is required for initial verification of PORD. In cases of severe loss of drug-metabolizing cytochrome P450 enzyme activities, further considerations about the clearance of drugs in patients with PORD would require evaluation. Effect of altered clearance of kidney transplant drug tacrolimus has been indicated in several studies among the people with POR*28 allele (Zhang et al., 2015).

## Data Availability Statement

The datasets generated for this study are available on request to the corresponding author.

## Author Contributions

Participated in research design: AP. Conducted experiments: SP, MV, SU. Contributed new reagents or analytical tools: NK. Performed data analysis: SP, MV, AP. Overall supervision of the project: AP. Wrote or contributed the writing of the manuscript: SP, MV, SU, NK, AP.

## Funding

This work was supported by the Swiss National Science Foundation (31003A-134926), the Novartis Foundation for Medical-Biological Research (18A053), and a grant from Burgergemeiende Bern to AP. The funders were not involved in the study design, collection, analysis, interpretation of data, the writing of this article or the decision to submit it for publication.

## Conflict of Interest

The authors declare that the research was conducted in the absence of any commercial or financial relationships that could be construed as a potential conflict of interest.
